# 2-(9*H*-Fluoren-9-yl)-4-(4-fluoro­anilino)-4-oxo­butanoic acid

**DOI:** 10.1107/S1600536813013779

**Published:** 2013-05-25

**Authors:** Tetiana Matviiuk, Michel Baltas, Zoia Voitenko, Marian Gorichko, Christian Lherbet

**Affiliations:** a National Taras Shevchenko University, Department of Chemistry, Volodymyrska str. 64, 01033 Kyiv, Ukraine; b Laboratoire de Synthese et Physico-Chimie de Molecules d’Interet Biologique, Paul Sabatier University, 118 route de Narbonne, 31062, Toulouse, France; c Université de Toulouse, UPS, Laboratoire de Synthèse et Physico-Chimie de Molécules d’Intérêt Biologique, LSPCMIB, 118 route de Narbonne, F-31062 Toulouse Cedex 9, France

## Abstract

In the title compound, C_23_H_18_FNO_3_, the tricyclic 9-fluorenyl system is approximately planar (r.m.s. deviation = 0.0279 Å). The N—C(=O) bond length is comparatively short [1.359 (3) Å], which is typical for such conjugated systems. The N atom has a planar configuration [sum of bond angles= 359.8°] due to conjugation of its lone pair with the π-system of the carbonyl group. In the crystal, a three-dimensional network is formed through N—H⋯O and O—H⋯O hydrogen bonds between the amide and carb­oxy­lic acid groups and carbonyl O-atom acceptors.

## Related literature

For the synthesis of various succinic anhydrides, see: Clar (1942 ▶). For biological studies on substituted succinimides, see: Carroll *et al.* (2007 ▶); Miller & Johns (1951 ▶); Patsalos (2005 ▶); Rankin *et al.* (1986 ▶). For the synthesis of substituted phenysuccinamic acids, see: Galustyan *et al.* (2000 ▶); Stephani *et al.* (2002 ▶).
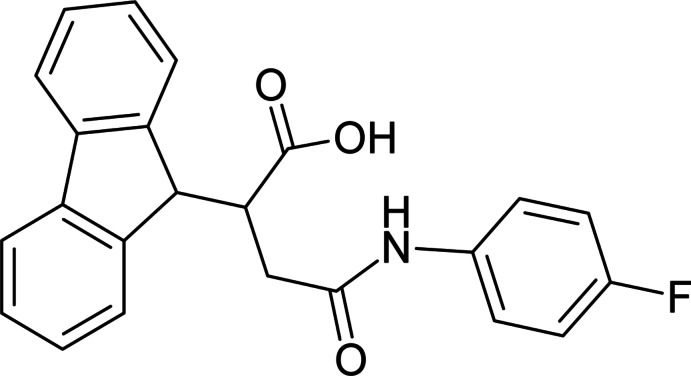


## Experimental

### 

#### Crystal data

C_23_H_18_FNO_3_
*M* *_r_* = 375.38
Monoclinic, 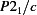

*a* = 10.2048 (6) Å
*b* = 18.5170 (11) Å
*c* = 9.6164 (6) Å
β = 90.494 (4)°
*V* = 1817.07 (19) Å^3^
*Z* = 4
Mo *K*α radiation
μ = 0.10 mm^−1^
*T* = 296 K
0.45 × 0.10 × 0.03 mm

#### Data collection

Bruker SMART APEXII CCD area-detector diffractometer
Absorption correction: numerical (*SADABS*; Bruker, 2008 ▶) *T*_min_ = 0.957, *T*_max_ = 0.997
8408 measured reflections
3205 independent reflections
1744 reflections with *I* > 2σ(*I*)
*R*_int_ = 0.081

#### Refinement

*R*[*F* ^2^ > 2σ(*F* ^2^)] = 0.054
*wR*(*F* ^2^) = 0.104
*S* = 1.00
3205 reflections
261 parameters
H atoms treated by a mixture of independent and constrained refinement
Δρ_max_ = 0.24 e Å^−3^
Δρ_min_ = −0.26 e Å^−3^

### 

Data collection: *APEX2* (Bruker, 2007 ▶); cell refinement: *SAINT* (Bruker, 2007 ▶); data reduction: *SAINT*; program(s) used to solve structure: *SHELXS97* (Sheldrick, 2008 ▶); program(s) used to refine structure: *SHELXL97* (Sheldrick, 2008 ▶); molecular graphics: *SHELXTL* (Sheldrick, 2008 ▶); software used to prepare material for publication: *SHELXTL*.

## Supplementary Material

Crystal structure: contains datablock(s) I, global. DOI: 10.1107/S1600536813013779/mw2107sup1.cif

Structure factors: contains datablock(s) I. DOI: 10.1107/S1600536813013779/mw2107Isup2.hkl

Supplementary material file. DOI: 10.1107/S1600536813013779/mw2107Isup3.cml

Additional supplementary materials:  crystallographic information; 3D view; checkCIF report

## Figures and Tables

**Table 1:** Hydrogen-bond geometry (Å, °)

*D*—H⋯*A*	*D*—H	H⋯*A*	*D*⋯*A*	*D*—H⋯*A*
O1—H1*O*⋯O2^i^	0.98 (4)	1.71 (4)	2.682 (3)	175 (3)
N1—H1*N*⋯O3^ii^	0.88 (2)	2.02 (3)	2.891 (3)	172 (2)

Symmetry codes: (i) 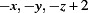; (ii) 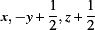.

**Fig. 1.**
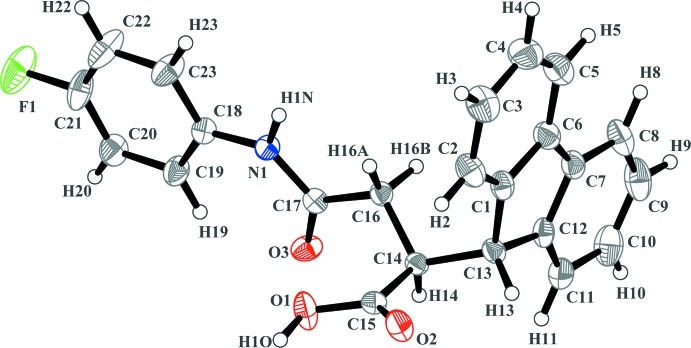
The molecular structure and atom numbering scheme for the title compound, showing 50% probability displacement ellipsoids.

**Fig. 2.**
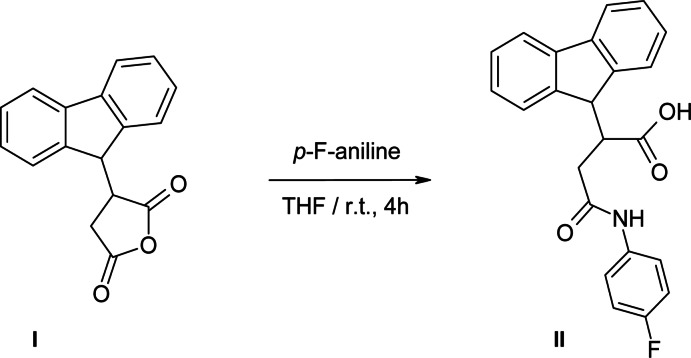
The synthetic route to the title compound (II).

## supplementary crystallographic information

### Comment

Derivatives of the pyrrolidine-2,5-dione fragment are common structural motifs in medicinal chemistry (Patsalos, 2005; Rankin, *et al.*, 1986). These molecules containing succinimide as a structural fragment were employed in drug design in response to their binding efficacy and low toxicity. Some pyrrolidine-2,5-dione derivatives were synthesized *via* interaction of succinic anhydride with different amines and futher cyclization, for example the synthesis of an nicotinic acetylcholine receptor antagonist (Carroll *et al.*, 2007). Cyclic anhydrides of dicarboxylic acids react readily with amines forming dicarboxylic acid monoamides (Miller *et al.*, 1951). Reaction of unsymmetrically substituted cyclic anhydrides may occur with formation of two possible regioisomers having the substituent either α or β to the amide group. (Stephani *et al.*, 2002.; Galustyan *et al.*, 2000). Herein, we report the regioselective synthesis and crystal structure of the title compound (II). The novel 2-(9*H*-fluoren-9-yl)-4-[(4-fluorophenyl)amino]-4-oxobutanoic acid, C_23_H_18_FNO_3_, (Fig. 1) is obtained as a product in the ring-opening reaction of 3-(9*H*-fluoren-9-yl)dihydrofuran-2,5-dione (I) (Clar, 1942) (see Fig. 2). The regioselectivity of the reaction depends on temperature. The reaction of anhydride (I) with *p*-F-aniline was carried out in dry THF at room temperature and a reactant ratio 1:1. Only one regioisomer (β-succinamic acid) was detected and isolated (91% yield). When the reaction was carried out at higher temperature (55 °C), a mixture of regioisomers was obtained.

In the structure of (II) (Fig. 1) the tricyclic 9-fluorenyl system C1—C13 is planar with an r.m.s. deviation of 0.0279 Å, wich is typical for this class of compounds. The N1—C17 bond distance is comparatively short (1.359 (3) Å) which is typical for such conjugated systems The N1 atom has a planar configuration, as the sum of bond angles on the N1 atom is 359.5 (17)°, due to conjugation of the lone pair of N1 atom with π-system of the carbonyl group. Molecules of compound (II) (Fig. 1) in the crystal are connected across a center of inversion by O1—H1···O2a hydrogen bonds forming dimers which are then connected into chains parallel to *c* by N1—H1N···O3b bonds (Table 1).

### Experimental

The synthesis of the cyclic anhydride (I) (Fig. 2) was carried out according to the literature method (Clar, 1942). Compound (I) (92 mg, 0.35 mmol)was dissolved in dry THF, *p*-F-aniline (39 mg, 0.35 mmol) was added and the mixture was stirred overnight at room temperature. Thereafter, solvent was evaporated and the residue dissolved in a saturated solution of sodium hydrocarbonate, filtered and acidified with 1 N HCl. The resulting precipitate was filtered off and recrystalized from ethanol. White powder, yield: 113 mg, 86%; m.p.: 171–172 °C. 1H NMR (300 MHz, [D6]DMSO, δ): 1.28 (d, J = 15.6 Hz, 1 H), 2.15 (dd, J = 11.4 Hz, J = 15.9 Hz, 1 H), 3.85 (d, J = 10.5 Hz, 1 H), 4.52 (br. s, 1 H), 6.90–8.05 (m, 12 H), 9.70 (br. s, 1 H), 12.86 (br. s, 1 H); 13C{1H} NMR (75 MHz, CD_3_OD, δ): 32.6, 44.3, 49.4, 115.8, 116.1, 120.9, 121.1, 122.8, 122.9, 125.4, 126.1, 128.1, 128.6, 128.8, 128.9, 136.0, 142.6, 143.1, 144.7, 146.0, 160.0 (d, J = 241.8 Hz), 172.5, 177.2. 19 F NMR (282 MHz, CD_3_OD, δ): = -116.0.

### Refinement

Carboxylic acid and amide H-atoms were located in a difference-Fourier synthesis and both positional and displacement parameters were allowed to refine. Other hydrogen atoms were positioned geometrically, with C—H = 0.96–0.98 Å and were allowed to ride on their parent atoms, with *U*_iso_(H) = 1.2*U*_eq_(methine or methylene C) or 1.5*U*_eq_(methyl C). In the absence of a suitable heavy atom, the absolute configuration of the title compound could not be determined.

### Crystal data

**Table:**

C_23_H_18_FNO_3_	*F*(000) = 784
*M**_r_* = 375.38	*D*_x_ = 1.372 Mg m^−^^3^
Monoclinic, *P*2_1_/*c*	Mo *K*α radiation, λ = 0.71073 Å
*a* = 10.2048 (6) Å	Cell parameters from 8408 reflections
*b* = 18.5170 (11) Å	θ = 2.3–25.0°
*c* = 9.6164 (6) Å	µ = 0.10 mm^−^^1^
β = 90.494 (4)°	*T* = 296 K
*V* = 1817.07 (19) Å^3^	Plate, colourless
*Z* = 4	0.45 × 0.10 × 0.03 mm

### Data collection

**Table:**

Bruker SMART APEXII CCD area-detector diffractometer	3205 independent reflections
Radiation source: fine-focus sealed tube	1744 reflections with *I* > 2σ(*I*)
Graphite monochromator	*R*_int_ = 0.081
ω scans	θ_max_ = 25.0°, θ_min_ = 2.3°
Absorption correction: numerical (*SADABS*; Bruker, 2008)	*h* = −12→11
*T*_min_ = 0.957, *T*_max_ = 0.997	*k* = −22→19
8408 measured reflections	*l* = −11→11

### Refinement

**Table:**

Refinement on *F*^2^	Primary atom site location: structure-invariant direct methods
Least-squares matrix: full	Secondary atom site location: difference Fourier map
*R*[*F*^2^ > 2σ(*F*^2^)] = 0.054	Hydrogen site location: inferred from neighbouring sites
*wR*(*F*^2^) = 0.104	H atoms treated by a mixture of independent and constrained refinement
*S* = 1.00	*w* = 1/[σ^2^(*F*_o_^2^) + (0.027*P*)^2^] where *P* = (*F*_o_^2^ + 2*F*_c_^2^)/3
3205 reflections	(Δ/σ)_max_ < 0.001
261 parameters	Δρ_max_ = 0.24 e Å^−^^3^
0 restraints	Δρ_min_ = −0.26 e Å^−^^3^

### Special details

**Table:**

Geometry. All e.s.d.'s (except the e.s.d. in the dihedral angle between two l.s. planes) are estimated using the full covariance matrix. The cell e.s.d.'s are taken into account individually in the estimation of e.s.d.'s in distances, angles and torsion angles; correlations between e.s.d.'s in cell parameters are only used when they are defined by crystal symmetry. An approximate (isotropic) treatment of cell e.s.d.'s is used for estimating e.s.d.'s involving l.s. planes.
Refinement. Refinement of *F*^2^ against ALL reflections. The weighted *R*-factor *wR* and goodness of fit *S* are based on *F*^2^, conventional *R*-factors *R* are based on *F*, with *F* set to zero for negative *F*^2^. The threshold expression of *F*^2^ > σ(*F*^2^) is used only for calculating *R*-factors(gt) *etc*. and is not relevant to the choice of reflections for refinement. *R*-factors based on *F*^2^ are statistically about twice as large as those based on *F*, and *R*- factors based on ALL data will be even larger.

### Fractional atomic coordinates and isotropic or equivalent isotropic displacement parameters (Å^2^)

**Table:**

	*x*	*y*	*z*	*U*_iso_*/*U*_eq_	
C18	0.0042 (3)	0.33919 (14)	0.9353 (3)	0.0216 (7)	
C14	0.2656 (2)	0.11015 (14)	0.9369 (3)	0.0202 (7)	
H14	0.2753	0.1104	0.8356	0.024*	
C15	0.1482 (3)	0.06320 (16)	0.9696 (3)	0.0229 (7)	
C17	0.1516 (3)	0.23277 (15)	0.8999 (3)	0.0203 (7)	
C13	0.3918 (2)	0.07796 (15)	0.9989 (3)	0.0214 (7)	
H13	0.3919	0.0254	0.9864	0.026*	
C11	0.5535 (3)	0.10772 (16)	0.7975 (3)	0.0319 (8)	
H11	0.5028	0.0842	0.7306	0.038*	
C19	−0.0347 (3)	0.35150 (16)	0.7993 (3)	0.0296 (8)	
H19	−0.0054	0.3213	0.7288	0.035*	
C7	0.5909 (3)	0.14513 (15)	1.0345 (3)	0.0259 (7)	
C20	−0.1172 (3)	0.40861 (16)	0.7674 (3)	0.0323 (8)	
H20	−0.1438	0.4170	0.6761	0.039*	
C2	0.3348 (3)	0.08065 (16)	1.2646 (3)	0.0330 (8)	
H2	0.2598	0.0526	1.2538	0.040*	
C4	0.4815 (3)	0.14928 (17)	1.4117 (3)	0.0413 (9)	
H4	0.5029	0.1676	1.4990	0.050*	
C1	0.4133 (3)	0.09618 (15)	1.1518 (3)	0.0236 (7)	
C16	0.2473 (3)	0.18896 (14)	0.9832 (3)	0.0202 (7)	
H16A	0.2193	0.1889	1.0794	0.024*	
H16B	0.3319	0.2128	0.9802	0.024*	
C12	0.5141 (3)	0.11031 (14)	0.9341 (3)	0.0231 (7)	
C23	−0.0407 (3)	0.38466 (16)	1.0388 (3)	0.0341 (8)	
H23	−0.0158	0.3766	1.1308	0.041*	
C21	−0.1584 (3)	0.45197 (17)	0.8716 (4)	0.0379 (9)	
C3	0.3702 (3)	0.10780 (17)	1.3944 (3)	0.0399 (9)	
H3	0.3180	0.0978	1.4708	0.048*	
C8	0.7078 (3)	0.17802 (16)	0.9971 (3)	0.0346 (8)	
H8	0.7591	0.2017	1.0633	0.042*	
C9	0.7465 (3)	0.17492 (17)	0.8601 (4)	0.0403 (9)	
H9	0.8251	0.1962	0.8342	0.048*	
C6	0.5273 (3)	0.13700 (15)	1.1689 (3)	0.0280 (8)	
C22	−0.1219 (3)	0.44173 (17)	1.0068 (3)	0.0441 (10)	
H22	−0.1514	0.4727	1.0760	0.053*	
C10	0.6708 (3)	0.14094 (17)	0.7615 (3)	0.0408 (9)	
H10	0.6980	0.1401	0.6695	0.049*	
C5	0.5615 (3)	0.16381 (16)	1.2995 (3)	0.0371 (9)	
H5	0.6373	0.1912	1.3112	0.045*	
O3	0.13680 (18)	0.22640 (10)	0.77394 (19)	0.0278 (5)	
O2	0.15591 (18)	0.00507 (11)	1.0272 (2)	0.0308 (5)	
O1	0.0355 (2)	0.09099 (11)	0.9269 (2)	0.0361 (6)	
N1	0.0878 (2)	0.28259 (13)	0.9779 (2)	0.0206 (6)	
F1	−0.23954 (19)	0.50859 (10)	0.83812 (19)	0.0604 (6)	
H1O	−0.038 (3)	0.0582 (18)	0.943 (3)	0.086 (13)*	
H1N	0.106 (2)	0.2840 (13)	1.067 (3)	0.025 (8)*	

### Atomic displacement parameters (Å^2^)

**Table:**

	*U*^11^	*U*^22^	*U*^33^	*U*^12^	*U*^13^	*U*^23^
C18	0.0223 (17)	0.0195 (18)	0.0232 (17)	0.0001 (14)	0.0031 (14)	0.0025 (14)
C14	0.0212 (17)	0.0185 (17)	0.0208 (16)	−0.0012 (13)	−0.0029 (13)	−0.0015 (13)
C15	0.0235 (18)	0.0241 (19)	0.0212 (16)	−0.0002 (15)	−0.0008 (14)	−0.0043 (15)
C17	0.0206 (17)	0.0203 (18)	0.0201 (17)	−0.0037 (14)	−0.0002 (14)	0.0041 (14)
C13	0.0202 (17)	0.0189 (17)	0.0249 (17)	−0.0007 (13)	−0.0062 (14)	0.0026 (14)
C11	0.0275 (19)	0.033 (2)	0.035 (2)	0.0073 (16)	−0.0026 (16)	0.0010 (16)
C19	0.0331 (19)	0.0319 (19)	0.0236 (18)	0.0101 (16)	−0.0028 (15)	0.0008 (15)
C7	0.0214 (17)	0.0211 (18)	0.0350 (19)	0.0025 (14)	−0.0050 (16)	0.0035 (15)
C20	0.036 (2)	0.034 (2)	0.0263 (18)	0.0095 (17)	−0.0052 (16)	0.0077 (16)
C2	0.0294 (19)	0.036 (2)	0.0334 (19)	−0.0054 (16)	−0.0049 (16)	0.0091 (16)
C4	0.052 (2)	0.043 (2)	0.028 (2)	0.0059 (19)	−0.0134 (19)	−0.0004 (17)
C1	0.0211 (17)	0.0223 (18)	0.0275 (18)	0.0015 (14)	−0.0009 (15)	0.0063 (14)
C16	0.0191 (17)	0.0213 (17)	0.0203 (16)	0.0005 (13)	−0.0008 (13)	−0.0002 (13)
C12	0.0201 (17)	0.0206 (17)	0.0285 (18)	0.0032 (13)	0.0008 (15)	0.0071 (14)
C23	0.050 (2)	0.032 (2)	0.0201 (17)	0.0133 (17)	0.0068 (16)	0.0042 (15)
C21	0.035 (2)	0.030 (2)	0.048 (2)	0.0173 (17)	0.0076 (18)	0.0156 (18)
C3	0.040 (2)	0.053 (2)	0.027 (2)	−0.0014 (18)	−0.0026 (17)	0.0085 (17)
C8	0.0218 (18)	0.036 (2)	0.045 (2)	−0.0044 (15)	−0.0080 (17)	0.0117 (17)
C9	0.0217 (19)	0.044 (2)	0.055 (2)	−0.0021 (16)	0.0040 (19)	0.0211 (19)
C6	0.0289 (19)	0.0236 (19)	0.0316 (19)	0.0000 (15)	−0.0056 (16)	0.0029 (15)
C22	0.066 (3)	0.036 (2)	0.031 (2)	0.0247 (19)	0.0153 (19)	0.0060 (17)
C10	0.033 (2)	0.049 (2)	0.040 (2)	0.0059 (18)	0.0094 (18)	0.0125 (19)
C5	0.033 (2)	0.038 (2)	0.040 (2)	−0.0025 (16)	−0.0096 (18)	0.0042 (17)
O3	0.0411 (14)	0.0281 (12)	0.0141 (11)	0.0038 (10)	−0.0010 (10)	−0.0011 (10)
O2	0.0260 (13)	0.0231 (13)	0.0430 (13)	−0.0037 (10)	−0.0082 (10)	0.0084 (11)
O1	0.0187 (12)	0.0319 (14)	0.0576 (15)	−0.0008 (11)	−0.0042 (11)	0.0148 (11)
N1	0.0266 (15)	0.0235 (15)	0.0116 (14)	0.0062 (12)	−0.0018 (12)	0.0010 (12)
F1	0.0747 (16)	0.0495 (13)	0.0572 (13)	0.0380 (11)	0.0059 (12)	0.0156 (11)

### Geometric parameters (Å, º)

**Table:**

C18—C19	1.383 (4)	C2—C1	1.385 (4)
C18—C23	1.385 (4)	C2—C3	1.390 (4)
C18—N1	1.410 (3)	C2—H2	0.9300
C14—C15	1.516 (3)	C4—C3	1.380 (4)
C14—C13	1.535 (3)	C4—C5	1.385 (4)
C14—C16	1.538 (3)	C4—H4	0.9300
C14—H14	0.9800	C1—C6	1.396 (4)
C15—O2	1.212 (3)	C16—H16A	0.9700
C15—O1	1.322 (3)	C16—H16B	0.9700
C17—O3	1.225 (3)	C23—C22	1.377 (4)
C17—N1	1.359 (3)	C23—H23	0.9300
C17—C16	1.497 (4)	C21—C22	1.362 (4)
C13—C1	1.522 (4)	C21—F1	1.373 (3)
C13—C12	1.522 (3)	C3—H3	0.9300
C13—H13	0.9800	C8—C9	1.380 (4)
C11—C12	1.378 (4)	C8—H8	0.9300
C11—C10	1.391 (4)	C9—C10	1.371 (4)
C11—H11	0.9300	C9—H9	0.9300
C19—C20	1.384 (4)	C6—C5	1.392 (4)
C19—H19	0.9300	C22—H22	0.9300
C7—C8	1.390 (4)	C10—H10	0.9300
C7—C12	1.396 (4)	C5—H5	0.9300
C7—C6	1.458 (4)	O1—H1O	0.98 (4)
C20—C21	1.353 (4)	N1—H1N	0.88 (2)
C20—H20	0.9300		
			
C19—C18—C23	119.1 (3)	C6—C1—C13	110.3 (2)
C19—C18—N1	124.5 (3)	C17—C16—C14	116.1 (2)
C23—C18—N1	116.5 (3)	C17—C16—H16A	108.3
C15—C14—C13	111.0 (2)	C14—C16—H16A	108.3
C15—C14—C16	112.7 (2)	C17—C16—H16B	108.3
C13—C14—C16	111.1 (2)	C14—C16—H16B	108.3
C15—C14—H14	107.3	H16A—C16—H16B	107.4
C13—C14—H14	107.3	C11—C12—C7	120.6 (3)
C16—C14—H14	107.3	C11—C12—C13	128.6 (3)
O2—C15—O1	122.7 (3)	C7—C12—C13	110.8 (2)
O2—C15—C14	123.8 (3)	C22—C23—C18	120.5 (3)
O1—C15—C14	113.5 (3)	C22—C23—H23	119.7
O3—C17—N1	123.8 (3)	C18—C23—H23	119.7
O3—C17—C16	123.5 (3)	C20—C21—C22	122.7 (3)
N1—C17—C16	112.7 (2)	C20—C21—F1	118.0 (3)
C1—C13—C12	101.3 (2)	C22—C21—F1	119.3 (3)
C1—C13—C14	113.7 (2)	C4—C3—C2	121.1 (3)
C12—C13—C14	112.1 (2)	C4—C3—H3	119.4
C1—C13—H13	109.8	C2—C3—H3	119.4
C12—C13—H13	109.8	C9—C8—C7	118.8 (3)
C14—C13—H13	109.8	C9—C8—H8	120.6
C12—C11—C10	118.7 (3)	C7—C8—H8	120.6
C12—C11—H11	120.7	C10—C9—C8	121.0 (3)
C10—C11—H11	120.7	C10—C9—H9	119.5
C18—C19—C20	120.3 (3)	C8—C9—H9	119.5
C18—C19—H19	119.9	C5—C6—C1	120.1 (3)
C20—C19—H19	119.9	C5—C6—C7	130.7 (3)
C8—C7—C12	120.1 (3)	C1—C6—C7	109.2 (3)
C8—C7—C6	131.6 (3)	C21—C22—C23	118.6 (3)
C12—C7—C6	108.3 (3)	C21—C22—H22	120.7
C21—C20—C19	118.8 (3)	C23—C22—H22	120.7
C21—C20—H20	120.6	C9—C10—C11	120.8 (3)
C19—C20—H20	120.6	C9—C10—H10	119.6
C1—C2—C3	118.7 (3)	C11—C10—H10	119.6
C1—C2—H2	120.6	C4—C5—C6	119.2 (3)
C3—C2—H2	120.6	C4—C5—H5	120.4
C3—C4—C5	120.3 (3)	C6—C5—H5	120.4
C3—C4—H4	119.9	C15—O1—H1O	112 (2)
C5—C4—H4	119.9	C17—N1—C18	129.5 (2)
C2—C1—C6	120.5 (3)	C17—N1—H1N	117.8 (17)
C2—C1—C13	129.2 (3)	C18—N1—H1N	112.5 (17)
			
C13—C14—C15—O2	−5.7 (4)	C14—C13—C12—C7	119.0 (3)
C16—C14—C15—O2	−131.0 (3)	C19—C18—C23—C22	−0.6 (5)
C13—C14—C15—O1	175.6 (2)	N1—C18—C23—C22	179.5 (3)
C16—C14—C15—O1	50.3 (3)	C19—C20—C21—C22	0.0 (5)
C15—C14—C13—C1	−82.6 (3)	C19—C20—C21—F1	179.7 (3)
C16—C14—C13—C1	43.7 (3)	C5—C4—C3—C2	−0.9 (5)
C15—C14—C13—C12	163.2 (2)	C1—C2—C3—C4	−0.2 (5)
C16—C14—C13—C12	−70.6 (3)	C12—C7—C8—C9	−0.6 (4)
C23—C18—C19—C20	0.2 (4)	C6—C7—C8—C9	177.5 (3)
N1—C18—C19—C20	−180.0 (3)	C7—C8—C9—C10	0.8 (5)
C18—C19—C20—C21	0.2 (5)	C2—C1—C6—C5	−1.4 (4)
C3—C2—C1—C6	1.4 (4)	C13—C1—C6—C5	175.8 (3)
C3—C2—C1—C13	−175.2 (3)	C2—C1—C6—C7	179.9 (3)
C12—C13—C1—C2	−179.8 (3)	C13—C1—C6—C7	−2.9 (3)
C14—C13—C1—C2	59.7 (4)	C8—C7—C6—C5	4.4 (5)
C12—C13—C1—C6	3.3 (3)	C12—C7—C6—C5	−177.4 (3)
C14—C13—C1—C6	−117.1 (3)	C8—C7—C6—C1	−177.1 (3)
O3—C17—C16—C14	−36.1 (4)	C12—C7—C6—C1	1.1 (3)
N1—C17—C16—C14	147.0 (2)	C20—C21—C22—C23	−0.5 (5)
C15—C14—C16—C17	−71.6 (3)	F1—C21—C22—C23	179.9 (3)
C13—C14—C16—C17	163.1 (2)	C18—C23—C22—C21	0.8 (5)
C10—C11—C12—C7	−0.5 (4)	C8—C9—C10—C11	−0.9 (5)
C10—C11—C12—C13	−179.5 (3)	C12—C11—C10—C9	0.7 (5)
C8—C7—C12—C11	0.4 (4)	C3—C4—C5—C6	0.9 (5)
C6—C7—C12—C11	−178.1 (3)	C1—C6—C5—C4	0.2 (4)
C8—C7—C12—C13	179.6 (2)	C7—C6—C5—C4	178.6 (3)
C6—C7—C12—C13	1.1 (3)	O3—C17—N1—C18	−5.9 (5)
C1—C13—C12—C11	176.4 (3)	C16—C17—N1—C18	171.1 (3)
C14—C13—C12—C11	−61.9 (4)	C19—C18—N1—C17	4.0 (5)
C1—C13—C12—C7	−2.6 (3)	C23—C18—N1—C17	−176.1 (3)

### Hydrogen-bond geometry (Å, º)

**Table:**

*D*—H···*A*	*D*—H	H···*A*	*D*···*A*	*D*—H···*A*
O1—H1*O*···O2^i^	0.98 (4)	1.71 (4)	2.682 (3)	175 (3)
N1—H1*N*···O3^ii^	0.88 (2)	2.02 (3)	2.891 (3)	172 (2)

Symmetry codes: (i) −*x*, −*y*, −*z*+2; (ii) *x*, −*y*+1/2, *z*+1/2.
